# “The pandemic only gave visibility to what is invisible”: a qualitative analysis of structural violence during COVID-19 and impacts on gender-based violence in Brazil

**DOI:** 10.1186/s12889-023-16675-8

**Published:** 2023-09-23

**Authors:** Luissa Vahedi, Samantha McNelly, Nina Lukow, Anna Carolina Fonseca, Dorcas Erskine, Catherine Poulton, Lindsay Stark, Ilana Seff

**Affiliations:** 1 Brown School, Washington University in St. Louis, 1 Brookings Drive, St. Louis, MO 63130 USA; 2 UNICEF Brasilia, Brasília, Brazil; 3 UNICEF Geneva, Geneva, Switzerland; 4 UNICEF New York, New York, USA

**Keywords:** Structural violence, Gender-based violence, Violence against children; Brazil, COVID-19, Food insecurity, Favela

## Abstract

**Background:**

The COVID-19 pandemic produced alarming rates of disease and mortality globally, yet few nations were as severely impacted as Brazil. The pandemic also exposed and exacerbated persistent forms of structural violence across Brazil, which complicated gender-based violence (GBV) prevention and response efforts. While structural violence is not new, the systemic pressure and uncertainty introduced by COVID-19 intensified the detrimental impact of structural violence on the lives of Brazilians impacted by GBV. This work qualitatively investigated how the COVID-19 pandemic amplified structural violence and GBV in Brazil.

**Methods:**

We analyzed key informant interviews (KII) conducted with 12 service providers working in sectors related GBV prevention and response in Roraima, Boa Vista, and Rio de Janeiro. Interviews were audio-recorded, transcribed, and translated from Portuguese or Spanish into English, before applying deductive and inductive coding approaches through a collaborative data reduction process. The theoretical lens of structural violence outlined by Farmer and Rylko-Bauer guided the thematic development.

**Results:**

Analyses identified three themes. First, structural violence manifests as policies of inaction and erasure, which reduce the opportunity for upward social mobility among GBV survivors including Black women, trans persons, and people who live in the favelas. Policies of inaction and erasure fail to acknowledge/adequately respond to the significant health and safety needs of these communities. Second, structural violence is a fundamental cause of violence against women and children. Finally, service providers described community driven responses that address the dire survival needs (i.e., food insecurity) imposed by COVID-19, within a context of structural violence. These community driven responses were innovative, agile, and based on dire needs expressed to, and observed by, the service providers interviewed.

**Conclusion:**

This analysis highlights how the COVID-19 pandemic exacerbated existing forms of structural violence prevalent throughout Brazil. Findings stress the urgency with which the Brazilian government and international organization must act to support community driven programs that strive to address the most basic human needs.

**Supplementary Information:**

The online version contains supplementary material available at 10.1186/s12889-023-16675-8.

## Background

Since the global expansion of COVID-19 in early 2020, Brazil experienced among the highest number of incident cases and related mortality globally, only trailing the United States and India [1, 2]. The intensity of Brazil’s COVID-19 caseload and mortality had severe and unintended consequences on populations marginalized by structural violence. Although COVID-19 first appeared in Brazil’s most developed municipalities and urban centers, it quickly spread to communities facing chronic and historic social deprivation due to structural violence [1]. Structural violence enforces and sustains violence against marginalized communities and remains largely invisible [3]. The concept of structural violence was first introduced by Johan Galtung, who described peace as the absence of both direct physical violence and indirect structural violence, which is linked to unequal power and resource distribution [4]. Farmer and Rylko-Bauer expanded this definition by naming specific social structures and institutions that contribute to the “violence of injustice and inequity” [3], including patriarchy, slavery, caste systems, colonialism, apartheid, neoliberalism, poverty, and harmful ideologies that sustain discrimination by race, ethnicity, gender, sexuality, and/or migrant status. Such social structures are described as violent because they result in avoidable death, illness, and injury [3]. Structural violence occurs on a continuum, linking invisible, normalized structural harms, like poverty and hunger, to more overt and visible harm, like intimate partner violence and femicide [5].

In Brazil, structural violence manifests as gender-blind and/or neglectful policymaking, stigmatization, a lack of essential infrastructure (i.e., water and sanitation), and neglected basic needs (i.e., food, water, shelter). Brazil’s favelas have been particularly subjected to structural violence. Favelas are informal urban settlements located within or on the outskirts of Brazil’s large cities. Growth and expansion of Brazilian favelas occurred during the rapid industrialization of the 1950-1970s, although favelas have existed since the late 1800s due to the rising cost of living in the city and the desire for workers to reside closer to their workplaces [6, 7]. Favelas are self-organized—and often self-reliant—entities; aside from the militaristic presence of the police, favelas largely operate outside the range of the State [8, 9].

Persons residing in favelas experience systemic State disenfranchisement and neglect; favelas are characterized by social deprivation, low income, and a lack of economic opportunity due the State’s lack of material and legal presence [10, 11]. Poor public health, medical care, and basic sanitation infrastructure, as well as high population density, made residents of Brazilian favelas particularly vulnerable to COVID-19 transmission and violence-related harms perpetrated by both organized crime and State security forces [10, 11]. For example, in Brazil persons working in the informal sector (i.e., cleaners, waiters, kitchen workers, day laborers, baggage handlers, etc.) were not eligible for COVID-19 support funds, meaning they were further disadvantaged than those in the formal, middle-class sectors [12]. Given that favela residents are overrepresented in low-skilled and low-paid labor, such policies enacted further structural harm regarding socio-economic losses during the COVID-19 pandemic [12].

The COVID-19 pandemic exacerbated the linkage between interpersonal and structural violence, perpetuating violence that is both visible and invisible, acute and chronic, individually felt and institutionally designed. Communities disproportionately affected by structural violence in Brazil include favela inhabitants but also additional populations such as migrants/forcibly displaced persons fleeing Venezuela, trans persons, and racialized minorities [1, 13, 14]. Such communities are subject to structural violence experienced higher COVID-19 incidence rates and heightened mortality rates, compared to Brazilians who were not socially vulnerable or living in neglected communities [1]. Migrants and other forcibly displaced persons from Venezuela, who already faced structural violence in their countries of origin, face additional structural social inequality as evidenced through lower wages and lower quality schooling and job environments [15].

Further, Brazil’s health and social service task-shifting to contain community transmission resulted in less attention paid to the protection needs of population facing structural violence, especially regarding gender-based violence (GBV) [16–19]. GBV includes any harmful acts (physical, sexual, psychological, or neglectful) directed at an individual based on their gender, typically rooted in gender inequality, the abuse of power, and discriminatory norms [20]. The increased incidence and severity of GBV during COVID-19 has been well-documented [16, 17, 21, 22] and follows the trends of previous infectious disease epidemics like Zika and Ebola [18, 19, 23, 24].

Prior the COVID-19 pandemic, Brazil faced a high burden of GBV. In Brazil, the lifetime prevalence of lifetime physical or sexual intimate partner violence among women of reproductive (15–49 years) age is estimated to be 23% [25] Brazil has a high and increasing prevalence of the most lethal form of GBV; with 4.7 femicides per 100,000 women in 2017 and a 20.7% increase in this number between 2007 and 2017, Brazil ranks 5th globally in femicides [26, 27]. As the country with the longest involvement in racial slavery in the Americas, Brazil continues to grapple with complex and institutionalized forms of racism and racial oppression which manifest for Black women in many ways, including discriminatory treatment and violence [28]. The burden, patterning, and perpetration of GBV is not random and disproportionately burdens marginalized communities and identities, including women and girls, trans individuals, Black individuals, favela residents, and migrants/forcibly displaced persons [29, 30]. Among femicide victims, Black women are overrepresented, with 65% of murdered women in 2017 being Black. Since 2007, the disproportional victimization of Black women has been growing, compared to non-Black victims [26]. Further, as a result of homophobic and transphobic ideologies, Brazil’s LGBTQIA + populations, including 2.9 million gay and bisexual, and 3 million transgender and gender non-binary people, also experience erasure, social exclusion, and premature mortality, including through homicides [31–33]. In fact, Brazil has more trans and queer victims of homicide than any other country [33, 34].

While GBV was already highly problematic in Brazil prior to the pandemic, COVID-19 reaffirmed these gendered and racial health disparities. Quarantine and isolation policies during the pandemic kept women in the homes with their abusers, limited their ability to work and earn an income independent from their abuser, and prevented survivors from being able to access GBV services [35]. The mounting household stress related to the pandemic, isolation and lockdown policies, and gender inequitable norms created a particularly fragile and dangerous situation for Brazilian women and children.

Analyzing GBV through a theoretical lens of structural violence will broaden our understanding of what constitutes violence and the fundamental causes of GBV. This, in turn, may benefit policymaking by reducing discriminatory ideologies and advancing system-level strategies, rather than individualizing phenomena. Using structural violence as an analytical lens, this qualitative study draws on the perspectives of 12 Brazilian GBV service providers to investigate the interplay between structural violence and violence against women and children, during the COVID-19 pandemic in Brazil.

## Methods

The analysis presented here employs data collected in Brazil as part of a larger, multi-country study that sought to explore GBV prevention and response during the COVID-19 pandemic from the perspective of service providers working in low- and middle-income countries or with forcibly displaced populations. The data collected in Brazil sought to examine these research aims as they relate to service providers working with marginalized women and girls, including those living in favelas, migrants/forcibly displaced populations, LGBTQIA + individuals, and racial minorities, among others. Comparable data were also collected in Italy (regarding service providers working with forcibly displaced populations), Guatemala, and Iraq, and findings from these sites have been published elsewhere [36–38].

### Data collection

For the global study, the research team developed one semi-structured key informant (KI) interview (KII) guide for each service provider type, in collaboration with UNICEF headquarters. This KII guide was then adapted for the Brazilian context, based on feedback from the UNICEF country office to add additional questions on sexual exploitation (which was of particular concern to UNICEF Brazil). Interview questions asked respondents about their perceptions of changes to women and girls’ safety during the pandemic, service provision challenges and innovations, providers’ mental health and wellbeing, and other topics. Interviews were conducted flexibly and probes were adapted based on the KI’s area of expertise. For example, probes were tailored according to the service provider type: cultural mediator, service provider (legal, medical, mental health, psychosocial services, police), and GBV hotline staff. The KII guide was translated from English into Spanish and Portuguese prior to implementing the interview.

KIs were selected for invitation into the study using purposive sampling to ensure participants reflected a range of sectors and organizational roles. Local UNICEF partners generated the list of invited participants based on knowledge of services in the study sites as well as existing partnerships. Potential participants were invited to participate in the study via e-mail. Seventeen KIs were invited to participate in the study; twelve individuals agreed to participate, and seven individuals did not respond to the invitation or were not available for an interview.

The 12 Brazilian GBV service providers operated within Roraima, Boa Vista, Maré (a favela located in in the north zone of in Rio de Janiero) and Rio de Janiero. Service providers working for governmental and non-governmental organizations were represented in the data and worked across a variety of sectors: survivor support group, police, forced migration, health care, anti-violence advocacy, psychosocial support, mental and psychological care, and child protection. The KIs included: a community mobilizer, two cultural mediators, a police delegate, three health social workers, a protection coordinator, a child protection case manager, an NGO director and executive director, a psychologist, and the director of a specialized police department. Interviews were carried out by a UNICEF staff member who was fluent in Spanish and Portuguese. Interviews were conducted by phone (nine interviews), phone and Skype (one interview), phone and Whatsapp (one interview) and in-person (one interview), based on the key informants’ desired modality. Ten interviews were conducted in Portuguese, one was conducted in a mix of Spanish and Portuguese, and one was completed in Spanish. Verbal consent was obtained prior to the start of the interview, and all interviews were one to two hours in length. Study procedures were approved by the Health Media Lab Institutional Review Board (#361GLOB21).

### Analysis

Interviews were audio-recorded, transcribed, and translated from Portuguese or Spanish into English by a professional company. Due to limited funds, no back translation into Portuguese/Spanish was performed. All identifying information, including names and locations, was removed from the transcripts. An analysis team, comprised of four individuals (trained by the lead author), employed both deductive and inductive approaches to develop a codebook, as per the approach outlined in Deterding and Waters (2021) [39]. The interviewer did not engage in data analysis. The team first divided all the transcripts among the analysts such that each analyst worked with three to four transcripts and some transcripts were duplicated between analysts. Each analyst read through their assigned transcripts, drafting analytical memos simultaneously. During this process the team considered previous codes included in a codebook that had previously been used in Guatemala for the same study [37]. The team reflected on how these codes did or did not apply to the Brazil data. The research team reviewed and discussed the analytical memos and then adapted the codebook from Guatemala accordingly. Examples of adaptations made to the codebook included: changes in child codes representing the types of GBV, as commonly mentioned forms of GBV differed between countries; addition of a new set of codes capturing the places and circumstances of vulnerability (i.e., favelas), as this emerged as an important factor in the Brazil context; and, a new code capturing instances of violence against children, including neglect.

Beyond the initial analytical memoing exercise and using all transcripts, the team continued to add, remove, and adjust codes by conducting coding practice sessions, codebook piloting/coding test sessions, and team meetings until a final draft of the codebook was agreed upon and each code represented a distinct unit of meaning (refer to Additional file 1). For example, following the independent memoing exercise, each team member prepared a list of data-driven codebook adaptations that were discussed and debated in a reflexive manner to develop the second version of the codebook. Following this, the team piloted the codebook to identify discrepancies in code application. The team met to discuss code application discrepancies as well as further refined codes and their definitions, thereby developing the third version of the codebook. Discrepancies in code application were resolved through consensus and refining the code definition until all team members had a shared understanding. Finally, prior to beginning the formal analysis, the team conducted a final test of the codebook by coding the same set of two transcripts and reviewing the results. Any remaining discrepancies were discussed and resolved through consensus and definition refinement. During the coding test session, the interrater reliability between the analysts suggested substantial agreement (Cohen’s Kappa values between all analysts were 0.69–0.73).

The 12 transcripts were divided among the team members, who were responsible for applying the final version of the codebook to analyze the dataset. Following coding of the full dataset in Dedoose [40], the team met to discuss prominent and re-occurring concepts reflected in the data and data displays were used to organize coded excerpts and identify emerging themes. Given the Brazil data focused on the socio-political context of inequities and environments of increased marginalization, the team concurred that the theoretical lens of structural violence was useful in interpreting the prominent and re-occurring concepts that emerged when coding as well as to conceptualize how concepts related to each other. In applying the theoretical lens of structural violence to the codes, we developed the final thematic structure and present this in the results section. To improve credibility, the research team maintained an audit trail of all analytical memos, codebook versions, and recorded meeting and corresponding notes.

## Results

### Overview of the thematic structure

Utilizing a structural violence lens, our results highlight how structural violence in Brazil manifests as policies of inaction and erasure, leading to violence against women and children. Structural violence disproportionately impacts the health and security of communities at the margins of Brazilian society: people living in favelas, Black and LGBTQIA + persons, and migrants/forcibly displaced populations. However, we also describe community-driven responses that strive to resist structural violence. We focus specifically on three themes – (i) structural violence manifesting as policies of inaction and erasure, (ii) structural violence as a fundamental cause of violence against women and children, and (iii) community-driven responses that resist structural violence – and describe these themes in relation to the challenges faced by GBV survivors and service providers during the COVID-19 pandemic. The themes subthemes presented below are summarized in Table 1.

**Table 1:** Summary of the thematic structure

Theme	Sub-theme
1. Policies of inaction and erasure	a) Harmful ideologies b) Inadequate provision of basic survival needs c) State neglect
2. Structural violence as a fundamental cause of GBV	a) Continuity and visibility of child abuse and neglect during COVID-19 b) Continuity and visibility of violence against women during COVID-19
3. Community driven responses	n/a

### Theme 1: Policies of inaction and erasure

We use the phrase ‘policies of inaction and erasure’ to describe policies that fail to protect and promote the further marginalization of communities facing structural violence. Such policies fail to respond to dire health and safety needs and erase opportunities for upward social mobility, thereby perpetuating structural violence. The harmful effects of policies of inaction and erasure were magnified during the COVID-19 pandemic. For example, GBV survivors faced increased food insecurity, poverty, and adverse socio-economic conditions throughout the pandemic, leading to worsened health and security disparities. We explain the theme of ‘structural violence as policies of inaction and erasure’ by highlighting (i) harmful ideologies that justify such policies, (ii) the State’s inadequate provision of basic survival needs and (iii) the State’s neglect of communities facing structural violence.

#### Harmful ideologies

KIs shared that harmful ideologies (i.e., transphobia, racism, and misogyny) created safety and survival disparities affecting marginalized communities. Some service providers described the dangerous impact of transphobia and racism on safety. When describing most survivors who seek health care services following incidents of intimate partner violence, one social worker working in Maré (favela located in Rio de Janeiro) shared:

“Generally, the great majority are Black women [who are the primary caretakers of their households] … and these are very serious situations. We also have a growing number of transgender women, who are also old, who come asking for food and talk about all the violence they suffer, violence of countless forms” (KII 5).

The double burden of food insecurity and violence among trans women were not isolated incidents, but rather reflect a broader pattern of invisible or normalized violence against trans populations:

“We are in a country that murders the most [transgender] populations. Even in the pandemic, we had around two hundred transgender girls murdered in Brazil. Brazil leads this ranking. So, both outside and inside, violence against our population has worsened and intensified even more” (KII 8).

Multiple service providers also described how harmful gender norms, such as the sexualization of women and the gendered division of household labor, affected women and girls’ quality of life during the pandemic. During stay-at-home orders, one provider noted how women were objectified and “only seen as…reproductive and domestic worker[s]” (KII 8). As such, providers believed women experienced inequality due to the demands of household labor and being viewed as “an object for [men] to relieve themselves all the time*”* (KII 8). Another provider highlighted the invisibility of these domestic roles to the point of naturalization:

“So, everything that this woman does, or that these women who arrive ill, do, is bad, and they feel guilty, because they can't take care of the children, and, at the same time, take care of the house, take care of the husband, take care of the chores and are with those children 24 hours, because it's the moment that the children do not go to school, and she can't do anything else, only that” (KII 5).

The harmful ideologies of transphobia, racism, and misogyny inform policies of inaction and erasure that perpetuate structural violence via the inadequate provision of basic survival needs.

#### Inadequate provision of basic survival needs

Most service providers emphasized that the dearth of government-implemented public services and social safety nets magnified the lack of rights/safety among GBV survivors during the pandemic. This created an environment where meeting even basic needs became difficult for survivors. Unmet essential needs (food, water, and basic sanitation) were reinforced by harmful ideologies that undermine the humanity of marginalized groups, including Black, trans, migrant, and favela communities. As one KI working with LGBTQIA + survivors in favelas stated, “we still need to advance in public policies, in inclusive and non-exclusive policies, to think about humanization…People are no longer humanized” (KII 8).

Specifically, multiple service providers referenced the dire food insecurity faced by GBV survivors and their families. Providers emphasized that alleviating survivors’ hunger was a prerequisite to providing GBV care. Thus, service providers absorbed a core function of the State –poverty and hunger alleviation– as part of their day-to-day responsibilities:

“You get up and have someone at your door asking for a basket of basic needs, or you arrive at a house to do a home visit, and the person has been unemployed for three months and can’t pay and does not have anything to eat…This was the reality that we encountered on a daily basis” (KII 8).

Another provider described women who would “show us an empty refrigerator, an empty closet, a house with nothing to eat. And this also created and still creates today a lot of anguish in the team” (KII 7).

State inaction regarding the provision of basic needs amplified the structural violence experienced by survivors and eclipsed the ability of service providers to deliver GBV services. As one service provider noted:

“They are the families in poverty, below the poverty line. They are those families that come, for example, to the place and ask: ‘I just want food. I do not care if I get beaten, if my husband drinks every day, and comes drunk and hits everybody. No, I want food.’” (KII 5).

Another provider expressed how household tensions stem from hunger, describing that “when there was nothing to eat, [parents] got stressed” and “yelled a lot at the children,” but “it’s not like that, it’s not their fault that they are hungry*”* (KII 11). Another provider working in Maré echoed these statements and directly connected the violence of hunger to the structural violence of State neglect, observing:

“What also needs to be clear here is that while we see many advances in policies, we [service providers working in the favelas and the favela inhabitants] are only pleading for existence. There, as I said from the beginning: existence. The right to basic sanitation.. The right to decent food, the right to schooling. If we do not think about these factors first, how am I going to think about protection networks for women…for gender issues? This is not what she is going to discuss here, she is going to get here, first, hungry.” (KII 8).

A provider also highlighted the financial dependence some female survivors had on their abusers even after separation; survivors were “left with nothing until there is a judicial order that [the perpetrator] must pay food, a pension. So, until they get this, what does she live on?” (KII 7).

While government aid was distributed to households during the pandemic, some providers felt this short-term intervention had limited impact for Venezuelan migrants and refugees. For example, one provider working in child and adolescent protection with Venezuelan migrants in Boa Vista shared:

“But who gets aid, you know? How far does this take someone out of poverty? I have families that live in [rental units], in 3x4 rooms, more than 15 people, children that don’t have documents, that never went to school and that are in a peripheral area, that is very far from the Operação Acolhida service [Brazilian military operation focused on the humanitarian assistance of Venezuelan migrants], very far from accessing these things, and that live and depend on odd jobs for their husbands…they came to me to ask how they could help themselves” (KII 6).

In some cases, structural violence extended beyond the lack of basic need provision to a more generalized sense of State neglect. Service providers mentioned State neglect specifically in relation to Venezuelan migrants and the favelas.

#### State neglect of communities experiencing structural violence

Service providers mentioned the State neglect experienced by migrants and refugees who are forcibly displaced in Brazil. One service provider noted the economic damage wrought on Venezuelan communities due to border closure policies in response to COVID-19 transmissions: “Now [with] closing all the businesses, Venezuelans that were improving their condition because they were managing to set up their own business to have a little autonomy, they lost that” (KII 6). The same provider also questioned the policy decision to close the borders, stating:

“The argument was to avoid spreading the virus, but there was no way, we kept entering through other alternatives. For me, this was a political measure, this issue of closing the border had nothing to do with [COVID-19]” (KII 6).

Another provider connected border closures and the subsequent loss of income during the pandemic to an increase in sexual exploitation among Venezuelan women, explaining that:

“Because the migration happened very close to the pandemic…there was a context of people fleeing from poverty, from the situation that Venezuela is going through, and many of them were not prostitutes or living from sexual exploitation there, when they arrived here they saw this as a possibility for survival” (KII 11).

Movement restrictions also impacted Venezuelan migrants, who could not access GBV services and resources. Structural violence was felt across borders, as the violence of unemployment and lack of resources drove families to migrate, only to face new forms of violence in Brazil:

“There is the closing of the border and there were reports of women who were raped in front of their children because they came alone, because you lost all your offpportunities in Venezuela itself and you must leave, but you leave alone as a woman” (KII 6).

The provider also mentioned the lack of staff needed to provide care for migrant survivors during the COVID-19 pandemic:

“You come here and there is no one to take you in, to support you, to give you a service after having been through this. Why is that? Because everything is closed…many women in the same situation as her, with children, and there aren’t enough professionals to take care of them” (KII 6).

A second example of State neglect are the favelas. One provider emphasized that the unmet basic needs of GBV survivors in favelas are rooted in State abandonment:

“Politics is always thought of by someone from the middle class. So, that's why, for example, we can't have public policy for the favelas, because the people in power are middle class people that will find what's best for us…We must, every day, create new strategies to live.*”* (KII 8).

The provider also expressed “we are still there, in the fight, resisting and existing so that we can put an end to this harvesting of poor, Black, and favela lives” (KII 8).

Another provider working in Maré perceived the police force to be the State’s primary engagement with the favelas: “The favela collectives wanted to help people, the people themselves, because the presence of the State in the favela is only as a police force. So, it’s violence*”* (KII 5). To further illustrate this point, one provider referenced the “Jacarezinho massacre” (KII 8), which occurred during the pandemic. This massacre was a police operation that resulted in the deaths of twenty-eight people within the Jacarezinho favela and occurred despite a previous pandemic policy to limit police engagement in favelas [41].

Survivors who sought GBV support experienced a paucity of services against a backdrop of diminishing safety. One service provider described how movement restrictions and prioritizing infection control during COVID-19 limited their abilities to connect with survivors:

“Gender violence was not reaching the units. This was a big concern, because, maybe, they could not even get there. There wasn’t prevention work anymore, there were no home visits where we could say: ‘Look, I noticed, this, this, and this, when I went to the [home visit]’” (KII 5).

The provider also noted how COVID-19 was prioritized over violence protection, stating:

“Because the cases of violence, they are already difficult to talk about, at a time when everything is emergency and everything is focused on COVID, on vaccination, on the reduction of the epidemiological picture of contamination” (KII 5).

A police delegate serving Rio de Janeiro commented on the general lack of social services amid movement restrictions, sharing “So, I think that not being able, in a situation where you are forced to ask for people’s confinement, not having a service, right? I think that structured services in the municipality were lacking” (KII 4).

Finally, one health social worker also noted that survivors living in favelas who often experienced homelessness and substance use, internalize the stigma they experienced:

“I have seen women saying: ‘I did not know that I, with this [bad] life I lead … I did not even know that I could access a service like this, without being humiliated, without being sent away from that service, that I can’t enter because I am not clean, I am dirty” (KII 5).

### Theme 2: Structural violence as a fundamental cause of GBV

The perpetuation of structural violence, via policies of inaction and erasure, amplified the largely unmitigated and pre-existing issue of GBV. Providers discussed that during the pandemic, forms of structural violence (lack of public services, food insecurity, and militarization) contributed to an inequitable burden of violence against women and children. Thus, the often ‘invisible’ or normalized forms of structural violence contributed to visible forms of violence.

#### Child abuse and neglect

Some service providers noted that sudden school closures compelled children to spend more time at home with abusive family members, increasing the risk for violence against children. A healthcare social worker from Boa Vista noted that “children were the least affected in relation to COVID-19, but, in a broader view, [were] the ones that suffered the most,” particularly regarding the risk of experiencing sexual violence in the home, where children “become easier prey for these predators” (KII 10). The sudden school closures deprived children and adolescents of material and social supports typically provided in the school environment; according to a child protection service provider, this contributed to “cases of [self-harm] …[and] the pandemic made this emerge more” (KII 12). Another provider shared similar sentiments, stating “what I've seen that has grown, is [the need to care for] children in suicide attempts” (KII 10).

Similar issues regarding school closures and lack of violence reporting were raised by a service provider from Boa Vista who coordinated care for women and child violence survivors:

We are now experiencing the schools receiving these children again. So, everyone is expecting– unfortunately, a very bad expectation –[that] cases will start coming to us, because we know that the school is a very big gateway [to reporting]. It is where, many times, the teachers, the educators can identify, they can [report]. (KII 9).

School and economic closures also intensified household stressors in a context with liminal social protections, thereby increasing children’s exposure to domestic violence between paretns:

Both [parents] had to stay at home, they couldn't get jobs, sometimes there are women and husbands who can't stand each other, and they stayed at home all day long. We saw a lot of domestic violence from both of them, because if this one yelled the other one yelled louder … screaming and yelling in front of the children (KII 1).

Providers stated that it was not only systemic school closures which placed children in potentially dangerous situations by removing them from resources, but also the neglect and abuse perpetrated by family members. For example, one KI in the child protection sector in Rio de Janiero shared:

“15 days that I talked to a teenager, and he cuts himself all over: arms, legs. He does it in the middle of the night. His mother is always fighting with him, you know? He tells me that his mother says: ‘Ah, I want you to die.’” (KII 12).

A cultural mediator working with Venezuelan migrants forcibly displaced in Brazil noted how the negative educational impact of school closures and the chronic under-resourcing of online education increased the risk for family neglect:

“The school[s]...went to an online format and all the assignments were sent by phone or they had to look for a workbook…How were they going to access the workbooks to do the assignments? Or, what if their parents didn't have a telephone? … or if they live in a broken family that doesn't give a damn about them, how were they going to study?” (KII 6).

Similarly, a KI working in the Roraima police force described a circumstance where:

“The mother really punished the child a lot because she couldn't keep up with the online classes, the [child] didn't have the profile to watch online classes and she couldn't, so she got upset and hit the boy a lot.” (KII 11).

The experiences shared by this provider illustrate the harmful linkages between limited resources for families, school closures, household stress, and violence against children. Providers also stated that neglect sometimes put children in positions that exposed them to further harm. For example, one a child protection specialist explained:

“In our area, we see a great number of girls on the streets. We have cases where the mother leaves the girls at the door of the market and they stay there all day long asking for money. We do not know if those girls had lunch… This is violence against that little girl” (KII 12).

Household economic vulnerability resulting from limited employment opportunities and lack of State-sponsored support often fueled these forms of child neglect. In circumstances of structural violence, neglect does not necessarily arise from parental apathy, but out of a need to secure basic survival needs:

“In this daily life, when they go out to work, which is the only way to take care of that family, of children, and then they leave the children alone. So, they are only pointed out as the woman who leaves the children alone and goes to work. But at no time does anyone listen to this woman, no one thinks that this woman does not have a support network. With whom will she leave her children? If she does not work, she will die of hunger with her children” (KII 5).

The pandemic also limited avenues for identifying and reporting instances of child abuse and neglect. School closures directly inhibited the identification and referral of child abuse cases. Further, a health care social worker from Boa Vista explained that even if a child reports being abused to a parent, the financial dependence that a mother may have on the perpetrator may limit her ability to effectively seek services: “there are those mothers who are financially dependent and end up…not believing and not seeking care, or only seeking care because of a complaint or because of a report from the girl's school” (KII 10). This demonstrates the importance of school-based reporting mechanisms, and how limiting children’s access to these reporting mechanisms during the pandemic may have contributed to fewer reports being made.

Service providers also noted that adolescent pregnancy and child marriage continued throughout the pandemic and that these phenomena were a direct result of ongoing structural inequalities faced by young women and girls. One provider, who worked primarily with Venezuelan migrants as a cultural mediator explained that:

“[Girl child marriage/early unions] comes from an absence of the State within the communities, in the families, there isn't a family planning orientation...it's a total loss of service. So, I have dysfunctional families that have daughters that become adolescents and go to the street and come [back] married…these cases, which are many, when I have adolescents here getting married...having relationships with older men and getting pregnant” (KII 6).

The same service provider specifically named poverty as another contextual factor in early pregnancy:

“Poverty, poverty, the same thing that generates teenage pregnancy in all...the peripheries, poor places, and finally, girls without access to education, without access to service, without access to guidance, to planning, to... find the only way to relate to each other through...sex and dating…And it's a cycle” (KII 6).

Service providers highlighted that sexual exploitation against women and girls intensified during the pandemic due to high unemployment and the lack of social safety nets:

“We see many women, even girls, we see teenagers going to the prostitution side…we have many girls that…allow themselves to be abused due to the situation they live in... We have cases where people in charge know that their daughters are there, but they do not make a point of trying to find out” (KII 12).

Another service provider highlighted how the pandemic intersected with the ongoing marginalization of Venezuelan migrant communities and substandard housing conditions to increase girls’ vulnerability to sexual violence:

“I think that the main thing is the migratory condition itself, right, that we are living, but the pandemic was in fact an attenuating factor…Look, she can't go to school and stays at home with men that, sometimes, are not your direct relatives...10 people living in a single house, in a single room, or in a single tent…this brought a huge increase in situations of sexual violence…intimate touches, (something we never had)...grooming, today we have many cases of grooming of minors” (KII 6).

#### Violence against women

In addition to violence against children, KIs also mentioned violence against women as a consequence of structural violence and policies of inaction and erasure. A member of the police force perceived that femicide occurred during the pandemic even among women who had pre-pandemic protective orders. This highlighted weaknesses in the judicial and police systems regarding the safeguarding of women’s lives during the pandemic:

“During the pandemic there were cases of femicide even though the victim already had a protective measure …I think there were two cases of femicide, where the victim already had the measure, right? And this happened during the pandemic. I do not know if maybe the system failed in this surveillance, because of the pandemic…I only know that it really happened during the pandemic” (KII 11).

Increased household stress due to reduced socio-economic opportunities was described as directly related to the perpetration of lethal violence, thereby linking the lack of economic protections to GBV. For example, one provider working with Venezuelan migrant populations described a situation wherein a husband murdered his wife after suspecting she was involved in sex work:

“Women… stayed at home taking care of the children. There was an increase of stress, of anxiety within the families…many fights were related to cases of cheating and possible prostitution of the woman… [one man] went about his daily life and came back and he didn't find his wife and ended up discovering that she seemed to be prostituting herself and ended up assaulting her. The case…that I ended up following up on later in the courtroom as an interpreter, which was a murder case” (KII 6).

Several providers also spoke of psychological intimate partner violence, which can pose particular challenges for both providers and survivors “because you have no witnesses,” and, “it's harder for you to prove psychological violence” (KII 5). A cultural mediator working for an anti-violence advocacy organization explained the consequences of psychological violence within a pandemic context:

“The person can’t express herself, can’t talk to anyone, sometimes this woman can’t even talk on her cell phone, can’t even call a friend to talk, she sometimes is even prevented from going to a psychologist, for instance, to talk, so it’s more complicated, I think, because of this monitoring, this presence 24 hours a day” (KII 3).

Providers highlighted how psychological violence remained significant throughout the pandemic. As described by a police officer during lockdown period, one woman arrived at a police station expressing her inability to continue coping with “a situation of a threat, in which the [perpetrator] put the gun inside the victim's mouth, pulled the trigger, and then said: ‘Look, this time it wasn't loaded, but next time it will be’” (KII 4).

For people who were already experiencing IPV before the onset of the pandemic, COVID-19 created new opportunities for physical and psychological victimization, particularly among those who had pre-existing health conditions. The new vulnerabilities introduced by the pandemic also pushed GBV systems to adapt their understanding of what constitutes violence and how to respond. One service provider explained a situation in which a woman’s abuser intentionally and unnecessarily took a prolonged bus ride to contract COVID-19, knowing that his partner had pre-existing conditions which made her vulnerable to infection. Upon returning from the bus ride, the abuser stated, “I will only leave here when all this is over or when you die” (KII 5). When recounting this incident, the service provider explained:

“This is already a very violent statement for us….We did case studies with the public defender's office about how this is violence and this is part of the domestic violence cycle because he knows where she is the most fragile. So, he did not actually hit her…And then, with all the orientation that was given by our legal department, she was able to get a protective measure from the court to keep him away from the home…So, this was something that we achieved. And that was something new, that we had never experienced” (KII 5).

Similarly, a service provider working in mental health advocacy for domestic violence survivors stated that “many of the women that we serve here, many have health complications” (KII 7), and even if the threat of COVID-19 infection was not explicitly employed as a violent tactic by abusers, the lack of attention to prevention and mitigation (in the context of the survivor’s existing vulnerabilities) also constituted a new form of violence. For example, the same provider stated:

“Many of the partners…did not follow the protocols…And we realized…She has a health problem and…he just goes to the bar to drink, does not wear a mask, comes home, sleeps with her and says that he does not have a problem…So, we also realized that this was violence. And these services were also able to bring other forms of violence that did not exist before, that were not visible before to the pandemic and to be also seen by the judiciary. In case of a protective measure” (KII 7).

In the context of COVID-19, psychological abuse and control manifested in a new way, as abusers sometimes dictated whether their partners and families could receive COVID-19 vaccination:

“We had a case of a husband who did not let his wife get vaccinated, nor his children, and they all ended up in the hospital, with COVID-19…and in serious cases, this husband passed away and the wife and children still recovering in the hospital. We also had another case in which a whole family could not get vaccinated because the father wouldn't let them…he was the male figure, and all the women, even with all the education, were silenced and could not receive any health care” (KII 5).

### Theme 3: Community driven responses

The COVID-19 pandemic not only impacted survivors’ needs, but also prompted service providers and civil society organizations to shift the way they approached services and advocacy. In many cases, organizations mobilized efforts to meet basic needs during the pandemic. Formalized services such as healthcare, police, and legal systems also reported innovations and changes to the way they responded to GBV. These community movements and innovations initiated in response to the impacts of COVID-19 did not come without cost; some service providers noted extreme personal sacrifice to continue their work in the face of this public health emergency.

To fill the critical food insecurity needs that underscored power hierarchies within abusive relationships, GBV service providers collecteddonated food baskets. In Maré, a favela in Rio de Janeiro, women’s collectives spearheaded actions against food insecurity:

“So, when the pandemic was at its peak, this peak of isolation, I was called by the favela movements, from Maré itself, the favela collectives, to work together with them on this issue of information about COVID-19, on the issues of food security, that many people stopped working and had nowhere to get money from, so they had nothing to eat, right?” (KII 5)

Providers also mobilized to help women support themselves through gastronomy courses and the provision of agroecological [urban farming] baskets. Notably, a healthcare social worker in the favelas described how, in response to observing the food insecurity around them, a community organization:

“Distributed…around 150 [urban farming] baskets, these baskets came with many products…and a booklet with explanations...So, all this was an articulation from favela to favela, the favelas working together. So, it is…a movement that has nothing to do with the State, nor with the municipality, nor with anything governmental. It is a movement of residents, activists, people from inside the favelas” (KII 5).

Another provider engaged in advocacy work in the favelas shared that her organization planned to start a gastronomy course for transgender women living in favelas. Service providers also shared that they responded to the shifting and intensifying food security needs of their clients by prioritizing food provision over GBV services. One service provider working in the favelas shared:

“During this period, we only delivered basic food baskets, we stopped all our activities. People needed to be fed…So, I think that all of us, the leaders, were caught up in a rethinking. So, the activities were suspended, and all the financial backers that we had, we tried to channel the resources we had into buying donations [of basic food baskets]” (KII 8).

However, in some cases, providers noted that shifting to broader hunger alleviation services helped to identify women and girls at risk of experiencing violence. As a result, providers were able to identify—though not always address—additional instances of GBV. For instance, one social worker would listen to women and girls’ lived experiences while distributing urban farming baskets:

I had to talk to each one of the women [recipients], and ask them if they had any questions, any problems...And then, the stories were of the most terrible possible violence: sexual violence, patrimonial violence, violence of being silenced all the time, physical violence, psychological violence…So, it is scary when you get to a group of women and create a bond, and a way of caring, you realize how great is the number of women who have been violated, from a very young age” (KII 5).

Another service provider described a similar shift to food provision during the pandemic, but also elaborated on the negative consequences of the chronic under-resourcing of NGOs, saying:

“[The food guarantee] hurt us, because even our organizations had to reduce the number of employees because we couldn't afford to pay them, because the resources were for the basic food basket donation. So, we were very fragile, weakened in this pandemic period, but we managed to cope and are recovering now. I think the problem is to think about the post-pandemic period. We are still not discussing this. Post-pandemic: how is this question of the survival of non-governmental organizations going to be?” (KII 8)

Nonetheless, service providers remained committed to maintaining GBV service continuity, often by relying on their own personal resources. A government social worker focused on anti-violence work highlighted challenges encountered during online service provision, mainly the dearth of sufficient technology and internet. To maintain GBV service provision, the team decided to use their own private phones:

“Because of the number of women who started to seek our service, that we would use our private phones…we did the following, the first-time appointments were all done by the institutional phone and the return by private phones. So, we had seven phones to be able to call women back, besides the two institutional phones. And this was supposed to work for three months, until the city could get new numbers for us. Unfortunately, we are still doing this today.” (KII 7)

Service providers also developed innovative partnerships with the private sector to help women meet basic needs. For instance, a police delegate spoke of an initiative wherein survivors who escaped abusive relationships were housed in safe and clean hotel rooms:

“A very cool project arose, which is a project with the [hotel] chain, which allows these women to be sheltered in the hotels of the chain, the closest one. So, it was something that, during this terrible moment, was something very good that emerged to help, and I think it is here to stay…While the public power does not get organized” (KII 4).

KIs felt that the lack of safety and survival needs experienced by under-resourced communities, such as those in favelas, occurred over multiple decades. The lack of a coordinated public service response during the COVID-19 pandemic extended policies of structural violence during a public health emergency. As explained by one service provider,

“We are talking about a historical period of denial of rights for the community in the favelas. The pandemic only gave visibility to what is invisible and showed how much we are an unequal country” (KII 8).

The same service provider also discussed how any existing support networks were made possible through civil society and dedicated community members, rather than governmental agencies:

“These support networks and these things that are built, are built by non-governmental organizations from civil society, which will think about how to keep these [peoples’] lives alive” (KII 8).

In favelas, it is the perseverance of dedicated but overworked advocates, rather than the State, that keeps the most vulnerable community members alive:

“To be in this position is to literally annihilate yourself: you have no social life, you have no personal life, you have no life. You live for the sake of the other. You are constantly thinking of new survival strategies” (KII 8).

Pandemic-related vulnerability and lack of State support may irrecoverably threaten the survival of civil society organizations providing life-saving GBV care in the favelas. The fundamental question that remains is how to strengthen civil society organizations by legitimizing their essential work through State funding and accountability structures.

## Discussion

Drawing on the perspectives of 12 GBV service providers working in Brazil, this study qualitatively examined the interplay between structural violence and violence against women and children in Brazil during the COVID-19 pandemic. Our analysis reveals how structural violence in Brazil underlines policies of inaction and erasure that led to the intensification of violence against women and children within the context of COVID-19, while also highlighting community-driven responses that strive to resist structural violence. Our analysis highlights that structural violence is a useful framework to connect GBV to social work and public health discourses around risk and protection, community development, and the social determinants of health.

KIs in our sample discussed their experiences serving communities in Brazil facing structural violence (favela inhabitants, trans persons, Black women, Venezuelan migrants). The increased food insecurity, limited employment opportunities, and school closures ushered in by the pandemic heightened household stress, leading to the amplification of violence against women and children. Structural violence via the legacy of Brazil’s policies of inaction and erasure facilitated the more pronounced impact of the COVID-19 pandemic on violence against women and children.

Our data highlighted pathways through which the State’s policies of inaction and erasure led to increased violence against children. For example, KIs described that when left with little to no social support, some women attempt to relieve household economic pressures through sex work, within a policy context devoid of robust protections that promote sex workers’ rights and livelihoods [42, 43]. Coupled with increased domestic responsibilities and economic pressures, engagement in precarious work such as sex work contributed to child neglect. In some cases, child abuse and neglect reflected severe economic constraints and lack of social protections within a pandemic context. Other scholars have also drawn attention to the deeper influences of structural violence on parenting and child abuse and neglect, emphasizing the challenging environment of poverty, inequality, and racism in Brazil [44]. Further, a retrospective cohort study from Brazil noted that for children under the age of ten and, women tended to be more frequently identified as the perpetrators of neglect [45]. However, as our analysis demonstrates, women’s involvement in neglect reflects the gendered division of child rearing labor and the lack of State-implemented social polies that support parents and women in child rearing during periods of elevated stress, such as the COVID-19 pandemic. For women facing structural violence, such as women living in favelas or Venezuelan migrant women displaced in Brazil, there is a greater level of State neglect and stigmatization that negatively impacts child rearing. Similar patterns have also been well-established in high-income countries; a systematic review found that income reductions, partly due to limited State assistance programs, led to increased future child maltreatment and neglect [46].

Further, pandemic-driven school closures in Brazil prevented children from accessing the most relied upon form of identification and reporting of child abuse and neglect [47]. While school closures were arguably necessary to combat the spread of COVID-19, this action combined with limited social programs created an environment where poverty increased and families were faced with the difficult choice of (i) leaving children unattended in order to work and provide food or (ii) supervising children while falling short of meeting basic needs. Policies and programs to improve household economic stability and provide essential food supplies may also reduce rates of child abuse and neglect in a pandemic context where schools are closed. These policies are especially lifesaving among communities subject to the structural violence of policies of inaction and erasure. Thus, social policies that improve case identification/referral in schools/communities, reduce food insecurity, and improve household economic resources are a value add for GBV risk mitigation. Local-level institutions (formal or informal), situated between the State and communities facing structural violence are well positioned to ameliorate survival needs and bolster investments in locally grounded movements for housing justice, food production, and neighborhood surveillance of child abuse/neglect.

Chronic underinvestment of community-driven organizations leaves women at risk of unique forms of violence in a pandemic context. KIs in our study repeatedly spoke to a range of incidents whereby psychological violence was perpetrated during the pandemic. In Brazil, psychological victimization is the most common form of violence perpetrated by intimate partners [48]. Given that psychological victimization can occur in the absence of any visible physical injuries, it is less likely to be recognized by victims, law enforcement, or service providers as warranting protection [49]. In our data, KIs mentioned situations wherein abusers capitalized on COVID-19-related fear and uncertainty to further exercise control [50]. KIs described that in some cases, abusers took actions to deliberately expose themselves to COVID-19, failed to take preventative measures to instill fear, or directly prevented victims (women and children) from receiving the COVID-19 vaccine. Similar forms of psychological violence within the context of COVID-19 have been reported in the empirical literature [51–54] and demonstrate dangerous mechanisms whereby abusers extend their power and control during stay-at-home orders and magnify infection and hospitalization disparities by gatekeeping survivors’ vaccination access.

### Implications

Given the structural violence faced by communities at the margins of Brazilian society (favela residents, trans persons, Black women, and Venezuelan migrants), policy changes are needed across a variety of stakeholders: women’s collectives, international donors, and the Brazilian government. Based on our analysis, we call on international organizations to strengthen the life-saving work and protection needs of women’s grassroots collectives, the Brazilian government to address dire food insecurity as a fundamental cause of violence against women and children, and civil society organizations to continue leading visibility campaigns that place human rights violations such as femicides and trans-femicides on the policy agenda.

Women marginalized by structural violence in Brazil, such as Black and trans women in favelas, have a long-demonstrated history of mobilizing for collective action. Over the last few decades, the country has witnessed a growth in critical consciousness around violence against women, and Black women, in particular. This increased political consciousness has engendered the organic establishment of women’s groups that provide mutual support and take lifesaving collective political action [55]. While McIlwaine and colleagues [49] called attention to the formal and reactive women-led networks that emerged in Brazilian favelas during the pandemic to address GBV and confer emotional support on survivors, KIs in our study also highlighted the critical role women’s collectives played in helping survivors meet their basic needs during the COVID-19 pandemic, filling in the gaps left unaddressed by public services. Studies from other contexts have also drawn attention to the critical role women’s collectives played in helping survivors and women meet their basic needs as well enabling households to buffer against the economic shocks of COVID-19 [56, 57]. While women’s collectives often form organically, more formal investment in these groups by both governmental and non-governmental agencies, including international donors, could help to improve survivor outcomes, mitigate harms engendered by psychological IPV, and increase the likelihood that women are involved in community decision-making processes. This local organization leverages the self-organization of favela inhabitants and does not impose State intervention, which has traditionally adopted a militaristic stance [41, 58, 59].

Our data also emphasizes a persistent and urgent need for government resources to alleviate food insecurity and malnutrition. While KIs discussed a range of health disparities in Brazil during the pandemic, such as survivors having an increased dependence on abusers, vulnerability to infectious disease, limited availability and access to GBV services, food security and malnutrition are underlying and fundamental causes of many threats to population health, therefore making food security an actionable policy target. The Universal Declaration of Human Rights, to which Brazil is a signatory nation, explicitly states that access to sufficient nutritious food is a basic right. Yet in the face of mounting poverty and a global pandemic, the Brazilian government focused on short-term and limited emergency aid payments to informal workers and their families, which did not adequately address the issue of mounting food insecurity [60, 61]. The Government of Brazil can supplement the budgets of social welfare agencies and ministries in order to alleviate the extreme hunger that disproportionately affected communities facing structural violence.

Brazilian civil society organizations have led visibility campaigns that decrease stigmatization of LGBTQIA + groups and place femicide and trans-femicide on Brazil’s human rights policy agenda. Advocacy and activist groups have also supported trans-led initiatives to increase awareness, such as the Transgender Day of Remembrance recognizing trans history and ancestors in Brazil [62]. Even with the 2019 criminalization of homophobia and transphobia [63], weak legislative implementation and highly conservative leaders have resulted in federal funding cuts to social support systems and increased prejudice in policing and criminal justice systems [64, 65]. Uplifting and listening to trans- and queer-led advocacy groups, researchers, and political candidates remains essential to increase LGBTQIA + visibility and human rights.

### Limitations

This research should be interpreted in light of some limitations. First, we interviewed only service providers. In-depth interviews with women and girl survivors might have introduced lived experiences of structural violence that were not mentioned by service providers. Second, access to stable internet connectivity was a prerequisite to participate in the fully remote semi-structured interviews, potentially excluding service providers based in rural and remote areas with limited internet access [66]. Consequently, this may have limited our ability to service providers working with remote Indigenous communities. We approached the data analysis in an iterative manner; however, we did not alter our selected participants in an iterative manner or deviate from the semi-structured interview guide based on participant responses. Although such iterative approaches to data collection may yield richer and increase data saturation, we did not opt to deviate from our a-priori analysis protocols. We also did not perform member checking, which may reduce the credibility of our findings. Further, transcripts translated into English were not back translated into Portuguese/Spanish due to limited funds. This may have reduced the quality of the translation. As the interviews were conducted in Portuguese and Spanish and the data were analyzed in English, it is possible that some of the language and meaning nuances were lost in translation. Lastly, our data did not speak to the long-term sustainability and resilience of community-driven responses that aim to resist structural violence. We encourage future research to investigate these topics.

## Conclusion

The COVID-19 pandemic and lack of social protection policies exacerbated existing structural violence in Brazil and increased the vulnerability of communities at the margins of Brazilian society: persons living in favelas, migrants, Black women, children, and trans persons. Throughout the pandemic, GBV service providers noted sharply increasing food insecurity which intensified other adverse outcomes, including worsening child abuse and neglect, emerging forms of psychological GBV, direct and indirect State -sanctioned violence, and deepening poverty. Even in the face of severe under-resourcing, service providers exhibited tremendous resourcefulness and dedication by shifting their service models to meet the most urgent needs. Our data highlights the lifesaving role that grassroots organizations play in directly serving and advocating for the most marginalized communities. To support GBV survivors and communities experiencing structural violence, the government of Brazil should promptly move to meet their obligations of providing adequate food security for all people in Brazil, civil society organizations should continue promoting visibility campaigns for the LGBTQIA + community and trans-femicides, and national and international donors should increase support and resources for grassroots women’s collectives.

### Supplementary Information

**Additional file 1.**

## Data Availability

The datasets used and/or analyzed during the current study are available from the corresponding author on reasonable request.

## Abbreviations

GBV: Gender-based violence
IPV: Intimate partner violence
KI: Key informant
KII: Key informant interview
LGBTQIA +: Lesbian, gay, bisexual, transgender, queer, intersex, asexual
NGO: Non-governmental organization
UNICEF: United Nations Children’s Emergency Fund
