# Supplementary material for: “The pandemic only gave visibility to what is invisible”: a qualitative analysis of structural violence during COVID-19 and impacts on gender-based violence in Brazil
Source: BMC Public Health. 2023 Sep 23;23:1854. doi: 10.1186/s12889-023-16675-8 (PMC10517463; doi:10.1186/s12889-023-16675-8)
Supplement: Supplementary file 1 — Additional file 1. [file 12889_2023_16675_MOESM1_ESM.docx]

Codebook

**Legend:**

Parent Codes are left justified.

- Child codes are indented using a bullet.

KI: key informant

KII: Key informant interview

VAWG: violence against women and girls

VAC: violence against children

| **Code** | **Description** | **Example*** |
| --- | --- | --- |
| PART A) Violence: Types and perceptions of changes | | |
| Violence Type | - KI discussing specific cases violence or reflecting on the phenomena more generally - Can be co-coded with: VAWG increased (inside the home, outside the home), VAWG decreased, Violence against children |  |
| - Child marriage | - Marriage or informal unions involving girls <18 years old - often mentioned in conjunction with a young woman/girl having a considerably older spouse/partner | “Q: Uh-huh. Now, Gilmara, I would like you to help me look at a more specific issue, which is the risk of early marriage and the risk of sexual exploitation. Do you think this has changed in your community since the pandemic started?  A: No. Girls are still having early marriages and they are still being exploited, sexually abused.”- #ID08-2_211014_163650- COMPLETA-PRONTO”  “I think that it is also the lack of, a lot of lack of information, regarding early marriage. We see our girls get involved and when the parents realize it, they can't save them anymore.”-#ID12_211104_161436-PRONTO |
| - Femicide | - Intentional murder of women because they are women or girls - Perpetrated by partners, ex-partners, family members, organized crime | A: “The only new thing that happened during the pandemic, here in Roraima, was that we didn't have victims of feminicide with a protective measure, and during the pandemic there were cases of feminicide, even though the victim already had a protective measure, and this was a new fact for us.  Q: As a disobedience, did the disobedience to the protective measures grow?  A: Yes. Unless I'm mistaken, I think there were two cases of feminicide, where the victim already had the measure, right? And this happened during the pandemic. I do not know if maybe the system failed in this surveillance, because of the pandemic, I do not know, I only know that it really happened during the pandemic.” - Entrevista_ID#11-PRONTO  “We are in a country that murders the most transvestite and transsexual populations. Even in the pandemic, we had around two hundred transgender girls murdered in Brazil. Brazil leads this ranking. So, both outside and inside, violence against our population has worsened and intensified even more.”-#ID08-2_211014_163650- COMPLETA-PRONTO |
| - Physical | - Consists of committing acts or other ill-treatment through the use of physical force, of a non-sexual nature, such as suffocation, strangulation, pushing, slapping, hitting, throwing objects at/near the survivor, etc. | She was at a party, her boyfriend arrived there with another woman and saw her at the party, he saw her at the party and grabbed her by the hair, put her in the car, hit her, broke her nails, (inint 01:35:52), she had some false nails, broke the nail until it reached the flesh. - Brazil_Boa Vista_#ID06-2_211013_150541 - COMPLETA-PRONTO |
| - Sexual | - Any sexual act, attempt to obtain a sexual act, unwanted sexual comments or acts aimed at exploiting a person's sexuality, using coercion, threats to harm or to use physical force, by anyone, regardless of relationship with the victim, in any setting, including transactional sex. - Includes: sexual assault, unwanted sexual touching, rape, marital rape. | “Yes, our focus is not only girls, you see? We gather... women... we had a situation of a child, but that the mother who was being raped by the father of that child, so we had to protect, besides that child, we had to protect that mother as well.” - #ID12_211104_161436-PRONTO |
| - Emotional | - Includes inflicting suffering or harm on emotional level, including coercion, threats, put-downs/name calling, guilt-tripping, shaming, forced isolation, verbal abuse, social exclusion, intimidation, humiliation, etc. | “But it's more in the sense of still basic violence, the psychological violence of our girls (inint 1:09:01) quite a lot. Let's say, sometimes even inside the house. That girl that when she decides to change her sexual option and the people responsible for her do not accept it, you know what I mean? It's still violence against our girls, you know. And sexual violence too.”- #ID12_211104_161436-PRONTO |
| - Psychological IPV | - Weaponization of COVD-19 - Gaslighting - Threats that make survivors always feel that their safety/wellbeing (or that of their children/dependents) is in jeopardy | “And we realized that it was something like: "She has a health problem and he is not doing anything. He just goes to the bar to drink, does not wear a mask, comes home, sleeps with her and says that he does not have a problem, he does not have any disease. So, we also realized that this was violence.”- #ID07_211014_112056-PRONTO  “But psychological and moral violence is more difficult to prove because you have no witnesses. And most of them were inside the house with their partners. And this is something that we have been seeing changing, because just like there are many campaigns for women to know their rights, at the same time these men are also starting to understand what violence is and what they can be held responsible for.”-#ID07_211014_112056-PRONTO |
| - Human trafficking | - Includes sex, labor, and child trafficking |  |
| - Digital | - VAWG or VAC perpetrated through information and communication technology - Could include sending threatening messages, calling/messaging repeatedly and in a harassing fashion, blackmailing with information/photos, unwanted explicit images, etc. |  |
| - Grooming | - Behaviors intended to build trust and rapport with a child (typically) in order to exploit that trust and vulnerability, often with the goal or perpetrating sexual abuse. - Anything related to seeking out and building relationships with young people with the intention of future exploitation | she can't go to school and stays at home with men that, sometimes, are not your direct relatives, people... 10 people living in a single house, in a single room, or in a single tent inside the Operação Acolhida, this brought a huge increase in situations of sexual violence. Sometimes we didn't have a specific case of sexual violence, but we had intimate touches, (something we never had)... we didn't have, we had intimate touches, (inint 21:23), grooming, today we have many cases of grooming of minors. -Brazil_Boa Vista_#ID06-2_211013_150541 - COMPLETA-PRONTO |
| - Violence against children (VAC) | - KI mentions or describes violence experienced by children <18 years (boys and girls) - Includes all types of physical and/or emotional ill-treatment, sexual abuse, commercial or other exploitation, which results in actual or potential harm to the child's health, survival, development, or dignity in the context of a relationship of responsibility, trust or power. - Includes solicitation of minors for sex work or grooming of minors for sex work - Inclusive of vicarious trauma/violence, such as children witnessing GBV within household | “Actually, I remember some occurrences when the pandemic was still going on, there was one that caught my attention, which was not gender violence, but was against a child, the mother really punished the child a lot because she couldn't keep up with the online classes, the boy didn't have the profile to watch online classes and she couldn't, so she got upset and hit the boy a lot. So, the pandemic brought a new context to look at, you must live with the person in the house for a longer period and these are other realities, and this really caused an increase in stress inside the homes, there was a lot of fighting, not only between husband and wife, the issue of coexistence in this way, but there was a lot of fighting between siblings, between father and mother, between mother and child, you know? That family context and many actions in the 190 in this sense, of various conflicts within the family.” - Entrevista_ID#11-PRONTO  No. I am not going to say that it has decreased. It has not decreased. We see many girls living on the streets, a lot of them. In our area, we see a great number of girls on the street signs. We have cases where the mother leaves the girls at the door of the market and they stay there all day long asking for money. We do not know if those girls had lunch.- #ID12_211104_161436-PRONTO |
| - Negligence/Neglect | D[epriving](https://en.wikipedia.org/wiki/Deprivation_(child_development)) a child of their [basic needs](https://en.wikipedia.org/wiki/Basic_needs), such as the failure to provide adequate supervision, health care, clothing, or housing, as well as other physical, emotional, social, educational, and safety needs | “As I said before, in truth, precarious care and neglect was what came out the most, and then came risk situations: 157 denunciations received. Living with drug users: 68 reports.”- #ID12_211104_161436-PRONTO |
| - Sex work | - Any mention of being paid for sex with money or goods/services - Can include survival sex - Can co-code with VAC for cases of child sexual exploitation and transactional/survival sex involving children | “Now, the sexual exploitation, let's say, the pandemic factor, we know that unemployment has become very high, so unfortunately, we see many women, even girls, we see teenagers going to the prostitution side, you see.”- #ID12_211104_161436-PRONTO |
| Violence against women/girls increased | - KI mentions that VAWG has increased as a result of the COVID-19 pandemic in Brazil. Also includes descriptions of how and why VAWG has increased within the context of COVID-19. - When specific types of VAWG are also mentioned, co-code with the specific type of violence: “Violence Type”. | “But what did we realize? Before the pandemic we had an average of five first time women per week, which is not much, it is very little. With the pandemic we reached a maximum of seven per day. So, we realized that the demand increased a lot, a lot. And in general, we tripled our attendance.”- #ID07_211014_112056-PRONTO  So, I think that these aggressions have occurred... have increased much more than before. We used to receive cases of violence, sexual abuse, child exploitation, but in this case now, we have increased...more, right?- #ID08-2_211014_163650- COMPLETA-PRONTO |
| - Violence severity/lethality | - References to the ‘seriousness’ of the violence committed, up to and including potentially lethal violence |  |
| - Inside of the home | - KI mentions or describes that VAWG inside home has increased within the context of the COVID-19 pandemic. - Inside of the home indicates that violence is perpetrated by family members, intimate partners, or housemates. - When specific types of VAWG are also mentioned, co-code with the specific type of violence: “Violence Type” | “Both people had to stay at home, they couldn't get jobs, sometimes there are women and husbands who can't stand each other and they stayed at home all day long, yes, here we saw a lot of domestic violence from both of them, because if this one yelled the other one yelled louder, but never all at once, but screaming and yelling in front of the children.” -BRAZIL BOA VISTA #ID_01-PRONTO |
| Violence against women/girls decreased | - KI mentions a decline of any type of VAWG inside and/or outside home since the start of COVID-19 pandemic. | “Look, in the beginning, we noticed that violence decreased, violence itself.”- #ID07_211014_112056-PRONTO |
| Women/girls as perpetrators of violence | - Women/girls as perpetrators of VAC, IPV or both - Could include womens/girls involvement in sex work as a facilitator or exploiter of other women/girls. | So, many times, in a situation in which the victim assaulted, to defend herself, scratches, tries to hold her arm, scratches the aggressor, this is often interpreted by the on-duty police officer as if it were a mutual injury, and then he understands that it is a case of opening an inquiry to investigate what really happened, instead of immediately arresting the perpetrator. So, these situations are very serious, because the woman goes to the police station, makes a register, and there is no concern with the fact that this woman will return home together with that aggressor. -- #ID04_211007_114808 |
| PART B: Places and Circumstances of Risk and Vulnerability | | |
| Favelas | - Any discussion of violence, particularly against women/girls or related to their unique vulnerabilities, but also inclusive of wider violence in favelas - Can include structural violence as it facilitates generally unsafe environments (lack of food, limited policy support/structures, limited infrastructure, limited education opportunities, etc.) - Also inclusive of favela collectives/movements created to protect against violence during COVID | “We are not just talking about a pandemic period; we are talking about a historical period of denial of rights for the community in the favelas. The pandemic only gave visibility to what is invisible and showed how much we are an unequal country. So, we face it on a daily basis, this is not just something that happened during this period. I think it is good to ratify that we are in another reality, it is another role. What is thought of in politics is thought of only in security, there is no support network, there is nothing. These support networks and these things that are built, are built by non-governmental organizations from civil society, which will think about how to keep these lives alive.”- #ID08-2_211014_163650- COMPLETA-PRONTO  “Existing and resisting within this space. We can't... that's it, I talked about the slaughter. We have a genocidal state, a government that is killing the poor favela dwellers. We do not even need to be intellectual to understand what is at stake. So, if we do not exist and do not resist within this territory, there is no point in thinking about other things.”- #ID08-2_211014_163650- COMPLETA-PRONTO |
| Other places of risk or vulnerability | - Locations that are described or mentioned in relation to violence or risk factors for violence - For example, borders, migration entry ports, bus stops in relation to Venezuelan migrants, violence shelters - Separate from favelas | “So, this was also something that we noticed that changed a lot during the pandemic, which is the fear of taking a bus and getting contaminated or suffering another violence or walking through an empty city and suffering another violence.”- #ID07_211014_112056-PRONTO  “And we also assisted others who were not only living with domestic violence, but also had jobs, but also experienced other forms of violence in their jobs. Moral harassment, threats, and unhealthy jobs. So, we ended up in this period of confinement accessing not only the issues of domestic violence, but all the other types of violence that these women have simultaneously experienced.” -#ID07_211014_112056-PRONTO |
| Living with or returning to aggressor | - Within the context of COVID-19 and related challenges, KI mentions factors that prevent a victim from leaving an abuser or what makes someone go back to a perpetrator - Including if they are forced to by other circumstances (shelters are full/no other places to stay, no finances to leave (especially regarding pandemic-related un or under-employment, etc.) | “We do not work with a basket policy, but seeing other women literally going hungry and saying: "I will go back to him because I can't go hungry with my son". So, this was a great challenge for us.”-#ID07_211014_112056-PRONTO  So, the aggressor would stay there, the woman would return with him, she would be transferred from the shelter, she would leave and go after her husband -Brazil_Boa Vista_#ID06-2_211013_150541 - COMPLETA-PRONTO |
| Socio-economic challenges due to COVID-19 | - Pandemic-related negative impacts on socio-economic status of women and girls, their families, their intimate partners, and communities more generally - Socio-economic challenges include negative impacts on income, education, and occupation - Includes: job loss, informal work, employment, exploitation, decreased education, increased domestic work, precarious labor. | “Within the daily routine of Family Health, we still have cases of fathers and mothers who lose their jobs, can't support their children, and, at some point, get sick, and without anyone noticing, without this person being able to ask for help and call the service, this person ends up committing suicide. So, it's a very serious situation of these crossings of violence as a context of poverty.” Brazil_RJ_#ID05_211011_143323-PRONTO |
| Food insecurity/malnutrition | - KI mentions women/girls and/or their families are facing food insecurity: Lack regular access to enough safe and nutritious food for normal growth and development and an active and healthy life. This may be due to unavailability of food and/or lack of resources to obtain food. - Within and beyond the COVID-19 pandemic - Also includes discussion or mention of malnutrition | “So, when the pandemic was at its peak, this peak of isolation, I was called by the favela movements, from Maré itself, the favela collectives, to work together with them on this issue of information about Covid, on the issues of food security, that many people stopped working and had nowhere to get money from, so they had nothing to eat, right?” Brazil_RJ_#ID05_211011_143323-PRONTO |
| Isolation | - Any discussion of individuals experiencing social or physical isolation as a result of COVID-19 and its impacts, positive or negative. - Note: social isolation being used as a form of abuse by a perpetrator would be coded as psychological violence. | “We often hear here that we miss being able to go out, being able to have the freedom to walk around and not be charged, you know? And this was already quite common before the pandemic, but afterwards I think that this pressure became greater, because now that we are at home, we kind of feel part of an environment, you know? We do everything inside our house, sometimes we think we do not need to go out, we do not need to do anything anymore, so I think this feeling of... we have talked with girls that are... the demand for psychologists is much higher and this lack of freedom, I think that is it.” Brazil_RJ_#ID03_211006_194721-PRONTO |
| PART C: Reporting and disclosures | | |
| Perceptions of Reporting/Disclosure during COVID-19 | - KI mentions or describes reporting behavior (to law enforcement or hotlines) pertaining to VAWG or VAC - Includes both increased or decreased reporting and reporting that remained constant | “So, what woman will arrive saying that she is there because she is suffering from violence and she wants an orientation, she wants help, she wants to denounce, she wants something to do with that violence and she can't talk in a triage, where there are innumerous people talking about other things of daily life. So, these cases will only come when they are in the doctor's office and can talk about it. They will go in there talking about something else, so that they can trust, have a bond with this team, to be able to talk to the team. Then, probably, the case will come to me. So, this reduced a lot, right? Because the cases of violence, they are already difficult to talk about, at a time when everything is emergency and everything is focused on Covid, on vaccination, on the reduction of the epidemiological picture of contamination.” Brazil_RJ_#ID05_211011_143323-PRONTO |
| - Disclosure to hotlines/services | - Services can include hospitals, psychosocial services, essentially disclosing or reporting incident of violence to anyone outside the legal sector/police - Disclosure for the purpose of seeking support or engaging with services | Besides psychologists? I do not know, I think that... as I said, I have never met anyone who has made a complaint, I have never talked to anyone who has made a complaint, an occurrence report, not from here in Maré, from outside I know. But I only know of women who really needed help and said they needed a psychologist, but I think that is it. When this violence happens directly, to talk about it directly, I think that only with psychologists. Brazil_RJ_#ID03_211006_194721-PRONTO |
| - Legal reporting | - Related to formal legal system to take legal action - Police |  |
| - Third party reporting | - When a report in response to violence is made by a person, other than the victim/survivor, who witnesses or learns about the violence |  |
| PART D: GBV System | | |
| Digitization of services | - When a key informant discusses their experiences or survivors’ experiences with GBV services/operations moving to remote/virtual formats - Experiences can be positive or negative - Can be co-coded with the digital divide (for example by gender), tech challenges, and/or innovative adaptations/new service | “It did not expand, and many of the institutions, they were closed. So, they were closed. So, this public, could only access one institution, a reference center, a specialized center, a specialized center for women's health, people could only access the professionals together, online. So, not everyone has access to the internet. Imagine a person who goes to get just food, does this person have access to the internet? Does he or she have a telephone? An illiterate person, how is he or she going to know how to use all that electronic stuff, right?” Brazil_RJ_#ID05_211011_143323-PRONTO |
| - Quality changes | - When a key informant discusses how a shift to remote/virtual formats impacts the quality, effectiveness, or content of provided services. - Can be positive or negative | In groups, we didn't manage to do it through virtual means, visits, we weren't doing it, so, it was just, really, listening to the telephone and we had to stay with what the person was saying there. So, in terms of effectiveness, I, as a technician, didn't feel as effective as we can see today. Joining, you know, the virtual service with the face-to-face service. We united the two and increased it. But only the individual one was not being satisfactory, as well as only the virtual one, for us, was not being satisfactory, because our follow-up could not be effective. And, today, we are starting to draw a new picture. In this almost post-pandemic period, we can envision another form of service in the Creas. #ID09_211019_111602-PRONTO |
| System coordination: Providers | - KI mentions or describes how COVID-19 has impacted system-wide coordination/fragmentation between discrete services: medical, legal, social (includes hotlines and victim assistance centers) - Includes providers navigating any component of the GBV prevention and response system while trying to deliver services to survivors | Yes. Look, this period of confinement was quite complicated, because not only did we not have access to these people, because we were not working with visits, or face to face access to these people, but we had to rely on reports that came in by phone. So, many times, we even needed to request that a video call be made and everything, but we had a lot of difficulty with the internet issue. Unfortunately, here in Roraima, we have many problems regarding the internet, both because of the electric power and because of the constant fiber breaks. Here, it rained, the internet goes down. So, we were very much in the dark, you know? We were very much in the dark, because we didn't really know what we needed to do, where we could send people at that moment when everyone was more closed. We didn't want to send the person out of the house to look for specific care, because we didn't know if they would find it, because of the chaos that the health system was in, you know? And, also, we... we felt confined, because we didn't know what steps we could take. #ID09_211019_111602-PRONTO |
| System navigation: Survivors | - System navigation refers to (1) how victim/survivors are able to navigate the medical-legal-system after disclosure/reporting AND/OR (2) their experiences in accessing the various forms of care/support they need, across the discrete sectors | “And before, they needed us to come to them. So, this could take one, two weeks, because the car had to be available, the team had to be available. All this contributed to the fact that the service took longer. And, today, because we can do this service over the phone, and, many times, even manage to send a referral, a document over the phone, it makes the access to this person much easier, because, instead of them coming here to get a referral to go somewhere else, we can, over the phone, exchange information and they can leave the call with their referral, knowing where they need to go... where they need to go, who they need to contact.” - Brazil_ Boa Vista_#ID09_211019_111602-PRONTO  “At first, they may come, but then, many times, due to the delay, due to the... due to the difficulty they feel in accessing other places, they end up becoming discouraged. So, I have heard... we have heard reports from women who said: "Ah, I don't even know why I denounced them. I don't even know why I started messing with this. It was good while it was quiet, because at least I could have my life. So, we have heard speeches like this. So, nowadays, we notice that the network is much more careful in getting this woman and going back to her, rather than her denouncing and running after her rights. She stops there when she denounces. She denounces and, many times, she regrets it. So, if it is not the network going after her to bring her to this context of understanding that it is her right not to live in a violent environment, she doesn't look for everything. Before the pandemic, it seemed that these women, they came much more after this service. After the pandemic, it seems that, -I don't even know if I can use this word-, but they seem to have gotten used to that cycle of violence. So, they come and go in a way that they don't really have recourse to any kind of monitoring, any kind of assistance. And we have even heard from women that they say that other women are in the cycle of violence because they want to be.” - Brazil_ Boa Vista_#ID09_211019_111602-PRONTO |
| Gridlocks | - Due to COVID-19, access to services across the medical-legal-social system is blocked or considerably slowed - Various factors can result in blockage: high demand, lack of human resources, service closures, etc. | “Actually, what I see is that we have a high demand and with the pandemic there was a lot of police officers, Covid, getting sick. So, this caused a delay in the procedures, why? Because we had to create a strategy to not stop attending to them, however, the procedure is not only the initial service, right? You must give continuity to that police investigation. So, there was no delay, of course, in requesting measures, making arrests, but in the conduction of the inquiry, why? Because there was a lot of sickness among the police, so I think this was a big reflex, and today we are still trying to put things in order, but it takes some time, because the pandemic was last year, it started at the beginning of last year. So, you are left with the remnants of what happened, to be able to put things back in order.” - Entrevista_ID#11-PRONTO |
| Government | Captures any mention or references to Brazilian government, both positive and negative. For example, wanting more partnerships with the government, mentioning corruption of the government, etc. |  |
| PART E: Challenges faced by service providers | | |
| Providers’ mental health | - Providers refer to the mental/emotional impact of COVID, resources available to them, including institutional polices. It can also include providers’ own experiences with COVID, their co-workers and family, and how such experiences impacted them. | “Guys, everything caused a lot of stress ((laughs)). In fact, just so you have an idea, I got sick, psychologically speaking, you know?” - Entrevista_ID#11-PRONTO |
| Provider’s fear/stress due to COVID | - When providers describe being afraid of contracting or spreading COVID-19 - Descriptions of COVID-19 related uncertainty inducing stress | “The biggest challenge I think was fear, right? Because people were very afraid of the moment. Because what would happen? The policeman would come to work during the pandemic, but he was very afraid of taking the disease home with him. So, for... in my analysis. Because the police procedure, we already have a certain amount of time as police, everybody is already used to it: "Ah, we are going to do something and I do not know what". But in the pandemic, you find yourself in a situation where you can't stop working, but at the same time you are afraid of contracting the disease, you are afraid of taking the disease into your home, then you come today, tomorrow you know that your colleague on duty is hospitalized, intubated, so this issue I think was the worst, of you having to return the next shift even knowing that you are vulnerable to this, I think it was the worst moment. - Entrevista_ID#11-PRONTO |
| Overall challenges faced by service providers during COVID | Challenges experienced in times of COVID related to how organization/team functions or professional challenges for respondent. |  |
| - Increased workload or work/life balance challenges | - When service providers describe higher caseloads or other professional responsibilities because of the pandemic | “The stresses were the number of demands, ((laughter)) the increase in the number of demands; the constant expectation that the workload would return to 40 hours, because it's one thing to spend half a period dealing with those demands and know that the other period you'll have a little more freedom to resolve your issues as well, and another thing, is that you'll be stuck all day with that demand.” Brazil_ Boa Vista_#ID09_211019_111602-PRONTO |
| - Budgets | - Budget/financial/human resource constraints. Budget considerations can include shortages of PPE, including face masks, gloves, glass shield, and cleaning supplies but also paying for/buying licenses for Zoom, personnel shortage. - Can be co-coded with human resource challenges | “Quite a lot. Because, in my case, as I was in the coordination position, what happened? I came every day, why? Because I had to change my team every day, because today so-and-so who was positive, is with Covid, so I had to take another police officer from another place and: "Come on, get over here". And so, every day was a big challenge, to keep the service running, so you arrive today: "Ah, so-and-so is with Covid, he's not doing well". - Entrevista_ID#11-PRONTO |
| - PPE | - Service providers reporting not having access to PPE due to lack of availability or supply chain issues - Could include challenges with clients not knowing how or refusing to properly use PPE when in contact with service providers | “And the only situation that was difficult was the acquisition of PPE, because the institution was not prepared, nobody was prepared, and we had to leave the policemen on the front line and then, we didn't have the necessary equipment, because what arrived was more for health, at that first moment. And so, it took time... it wasn't that it took a long time, but it took a little while for us to adjust and get the protective equipment, until things started to flow.- Entrevista_ID#11-PRONTO |
| - Tech challenges | - Issues with internet access or proper functioning, access to necessary technology and devices, service providers lacking necessary digital literacy | “Yes. Look, this period of confinement was quite complicated, because not only did we not have access to these people, because we were not working with visits, or face to face access to these people, but we had to rely on reports that came in by phone. So, many times, we even needed to request that a video call be made and everything, but we had a lot of difficulty with the internet issue. Unfortunately, here in Roraima, we have many problems regarding the internet, both because of the electric power and because of the constant fiber breaks. Here, it rained, the internet goes down. So, we were very much in the dark, you know? We were very much in the dark, because we didn't really know what we needed to do, where we could send people at that moment when everyone was more closed.” - Brazil_ Boa Vista_#ID09_211019_111602-PRONTO |
| - Human Resources | - Operational shifts due to decreased staff (i.e. due to sickness) or shifts that are impacted by removal of or lack of funding. | Actually, what I see is that we have a high demand and with the pandemic there was a lot of police officers, Covid, getting sick. So, this caused a delay in the procedures, why? Because we had to create a strategy to not stop attending to them, however, the procedure is not only the initial service, right? You must give continuity to that police investigation. So, there was no delay, of course, in requesting measures, making arrests, but in the conduction of the inquiry, why? Because there was a lot of sickness among the police, so I think this was a big reflex, and today we are still trying to put things in order, but it takes some time, because the pandemic was last year, it started at the beginning of last year. So, you are left with the remnants of what happened, to be able to put things back in order. Entrevista_ID#11-PRONTO |
| Prioritizing COVID-19 over GBV and forced displacement |  | the migration of Venezuelans was not so important, we were still suffering in Venezuela, we were still suffering inside the shelters, the situation continues, but people forgot that. It wasn't important anymore, so they didn't pay so much attention to the situations that were happening and it was a huge struggle for them to understand that it was important, because people didn't: "No, only Covid is important. To find medical attention, there isn't any, only Covid is important. Everything was very complicated. - Brazil_Boa Vista_#ID06-2_211013_150541 - COMPLETA-PRONTO |
| PART F: Challenges faced by victims | | |
| Deterrents of leaving abusive partners or reporting | - KI mentions or describes reasons why victims are unable to report, leave or flee from abusive relationships - Various factors are involved: economic constraints, coercion/manipulation/psychological abuse, lack of transportation services, unaware of the violence being perpetrated, lack of services, custody issues with children or pets, etc… | Yes, but what I am saying is that when the victim is a child or an adolescent victim of sexual abuse, regardless of the pandemic, it was already observed that the offenders and the crimes occur within the home, in most cases, it is either a stepfather, a neighbor, a brother, or someone who lives with that person. Now, not in the case of adult women, in general, why? Because it is not common practice in Brazil for married women to register rape against their husbands, even though we know that it occurs, but it is not common practice for married women to register, the registration occurs of girls, of women that are raped, and in the case of adult women this rape generally occurs outside of the home. Entrevista_ID#11-PRONTO  Here what happened? Because the migration happened very close to the pandemic, so the sexual exploitation here, it went to an extreme, because there is a neighborhood where many Venezuelan women started to prostitute themselves, mainly Venezuelan. In fact, there were practically 100% Venezuelan women there, and this prostitution was going on 24 hours a day. There is even a... that they used to call, that the program was 80 reals, so they started to call those people who prostituted themselves in that place the eight hundred. So, I can't say that it was due to the pandemic, the migration and the pandemic came together and then the flow gained an enormous proportion. Huge. And there was a context of people fleeing from poverty, from the situation that Venezuela is going through, and many of them were not prostitutes or living from sexual exploitation there, when they arrived here they saw this as a possibility for survival. So, the context of Roraima ended up being modified also by migration. Entrevista_ID#11-PRONT |
| Mental health of victims | - KI comments/reflections on the mental health status of women/girls, families, or other members of the community that are not service providers. - Includes fear of COVID-19 and contagion - Does not apply to the mental health of the KI. | “Before Covid, I think that the women had an outlet - as I was saying. Although they had these emotions before, I think that now everything is intensified, because of us being in this... I do not know if I can say pressure, but in this situation that we must live in this environment and everything is fine, you know? Staying at home always, we must understand that it is a moment, anyway. And then we end up internalizing this and thinking that this is common, but it turns out that these reactions, all this that even happened before of anxiety, nervousness, agony, these things, but now everything has intensified, because we think that it is normal at this time, but as we can't handle everything.” Brazil_RJ_#ID03_211006_194721-PRONTO  “If someone says: "Oh, I am fine, everything is fine". I am even suspicious of this person, because everything is not fine. So, people started to call the health services in crisis, many anxiety crisis, depressive symptoms worsening, and then, women in particular, already with all the issues brought before the pandemic, which was already gender violence, which was already the innumerous types of violence, and they already sought the services to take medication. Because that is it, in fact, these are not the women who demand, but the only thing that sometimes a doctor, a physician, can do in the service, when they can listen, is to medicate.” Brazil_RJ_#ID05_211011_143323-PRONTO |
| - Suicide/self harm | - Discussion of anyone harming or attempting to harm themselves in any way | “There are what? 15 days that I talked to a teenager, and he cuts himself all over: arms, legs. He does it in the middle of the night. His mother is always fighting with him, you know? He tells me that his mother says: "Ah, I want you to die.”- #ID12_211104_161436-PRONTO |
| Sexual and Reproductive Health | - KI comments/discusses VAW that violates sexual and/or reproductive health - Includes violence against mothers/pregnant women, birth control access, pregnancy pressure, etc. | “For example, a pregnant woman was doing her prenatal care and all her documents were burned by her partner. So, this is a very serious issue, because she is in need of care, of affection, of a look, and, in fact, she is suffering violence there, including patrimonial violence.” Brazil_RJ_#ID05_211011_143323-PRONTO |
| Digital Divide |  | not everyone has access to devices to be able to access this service. Inside the shelter we offered radio so they could listen to the radio and (inint 29:41) so they could watch it on TV, but what about the children or the adolescents that didn't go to (inint) because they didn't get a radio, by shelter, the ones that... the children that... the parents that don't have the service that Operation Acolhida offers and that don't have to buy a phone, they (consult) no kind of service, nothing. -Brazil_Boa Vista_#ID06-2_211013_150541 - COMPLETA-PRONTO |
| PART G: Nuance | | |
| Intersectionality | - KI reflects in the intersectional dimensions of VAWG and VAC or other issues related to COVID-19 - Intersectionality refers to the interconnected nature of race, class, gender, age, etc. that create overlapping and interdependent systems of discrimination/disadvantage - Includes reflections about Indigenous Brazilians | “Look, the groups of women that can access our services are elderly women, immigrant women, women with physical disabilities that are not hearing or speech impaired, because we don't have a Libras interpreter, so, this group would be out. And it wouldn't just be women, it would be any aspect: teenagers, teenagers serving alternative measures; children, too, we don't have people qualified for this follow-up with the deaf. Immigrants, we can do this monitoring, but with a lot of difficulty in relation to language, some people are calmer to understand and speak Spanish, but we have already accompanied Haitian families, and we were not able to speak Haitian Creole, we needed help from Google, and communication became very difficult. So, immigrants, in quotes, Ok? Not all types of immigrants we can serve. Including, it is also a demand from the team, Ok? That the team can take courses in other languages, because, wanting it or not, we end up receiving this public here.” - Brazil_ Boa Vista_#ID09_211019_111602-PRONTO |
| - Disability | - KI reflects on how violence and COVID-19 affects persons living with physical or mental disabilities | Today, we have 197, and 197, the person calls, but it is not an emergency, it is the idea of calling to report a situation where a woman is temporarily deprived of her freedom, a woman who, for example, is unable to move around, or due to a disability, or for a momentary issue. So, she can register by... by phone. We register over the phone, but it is still a very artisanal thing. -Brazil_RJ_#ID04_211007_114808-PRONTO |
| - Adolescents | - KI reflects on how violence and COVID-19 affects adolescents who are approximately 10-19 years old | I have in the shelter 1,500 people, in which there are about 300 or so children and adolescents. They are children who are not doing anything, not going to school, not going to the plaza, not going to... not going to anything, just there. And today we are managing to get other services at night, through other organizations. We... we have swimming, as I told you, carpentry, we have chess, other organizations (inint 03:43) service, before, inside the shelter, before the pandemic, we could have crowds, so we did sports championships. So, what changed during the pandemic? We had nothing, we had no access to the squares for the girls because the squares were already closed, we had no (inint 04:03), no swimming, no woodwork, no chess, none of that, no therapeutic group, we couldn't gather inside the shelter. So, they (inint)... they had reduced a little bit the opportunities. Then, I had the idle girls. -Brazil_Boa Vista_#ID06-2_211013_150541 - COMPLETA-PRONTO |
| - LGBTQ+ | - KI reflects on how violence and COVID-19 affects the LGBTQ+ population in Brazil | We also have a growing number of transgender women, who are also old, who come asking for food and talk about all the violence they suffer, violence of countless forms. -Brazil_RJ_#ID05_211011_143323 |
| - Race | - KI reflects on how violence and COVID-19 affects Afro-Brazilian, Indigenous, or other non-white populations |  |
| - Indigenous | - KI mentions or references Indigenous persons in Brazil |  |
| Migrants/Forced Displacement | - Any comments or discussions related to migrant or forcibly displaced populations within Brazil | The border was closed and this created a huge impact in the community, a lot of anxiety because of the estrangement of the family and this issue of the trochas, the border was closed and people started to enter illegally because when you immigrate in a forced way, you are not migrating because you want to, you will continue migrating because you don't want to migrate, you are forced to go through that, so, people started to create alternative methods to enter and we continued receiving families, without documents. -Brazil_Boa Vista_#ID06-2_211013_150541 - COMPLETA-PRONTO |
| - Haitians | - Forced displacement of Haitians | Immigrants, we can do this monitoring, but with a lot of difficulty in relation to language, some people are calmer to understand and speak Spanish, but we have already accompanied Haitian families, and we were not able to speak Haitian Creole, we needed help from Google, and communication became very difficult. So, immigrants, in quotes, Ok? Not all types of immigrants we can serve. -Brazil_ Boa Vista_#ID09_211019_111602 |
| - Venezuelans | - Forced displacement of Venezuelans | By then the situation was that the Venezuelan community was still entering, but through the "trochas", through alternative means, and there was no migratory regularization and then continued without access to the services because, first lockdown, service stopped, second semester, no documents, so, you can't access the services if you don't have documents. And the difficulty remained the same, but our efforts were not only to sensitize the community about the pandemic and to give an answer to the anxiety that the community was having because of the closing of the border and we only anticipated the serious cases, then we put cloths over the situations inside the shelter, put out fires.-Brazil_Boa Vista_#ID06-2_211013_150541 - COMPLETA-PRONTO |
| Moving Forward | | |
| Recommendations | - When participants provide ideas or perspectives for improved GBV services (availability, access, quality, effectiveness, policies, etc.) - Can also be used if coder thinks strategy discussed makes for an excellent recommendation for other providers |  |
| Innovations | - Any strategies initiated or adapted during the pandemic that relate to GBV prevention and response - Can be high tech or low tech, even a unique/different way of conceptualizing or organizing or coordinating with other organizations - Does not include digitizing service provision (use other code) | Saw campaigns on WhatsApp, in which people who needed help would hang a handkerchief in the window, the red-light campaign, that sign with the hands somewhere, in a pharmacy. So, I think that these campaigns did not have very good results, but I think that this possibility has grown a lot, and we are starting to see the press and the social networks - which I think play a very important role in the dissemination of this theme -, making people more concerned about paying attention to someone who is close to you, who might be living a situation of violence. And I think that the victims themselves also became more motivated to seek help. Sometimes I receive requests through Instagram. - #ID04_211007_114808  I assist women and children who are victims of violence in general, and recently I have also started to assist children and adolescents victims of sexual violence. So, I already know which are my cases, the neighborhood where I follow the most victims of sexual violence, for example, and then I share them with the other technicians here and ask them: "Such and such a neighborhood, how is it going for you? Then they say: "Look, there are so many children being followed up because of this in this neighborhood". So, our mapping is kind of internal, you know? So that we can develop our intervention and prevention strategies, you know?  - #ID09_211019_111602 |
| Benefit or silver lining | - Benefit can be related to availability, access, quality, effectiveness | “A: Look, now, I believe that finding, locating these people who are survivors, who are victims, it has become easier because we have this access through the internet. Before, as we could only call through the phone number of the institution, it was only recommended to call through the phone number of the institution, many times, we were not able to locate these families, because the address was wrong, the phone number was also wrong, sometimes the person had already changed the phone number, so, we were not able to locate them. The main comparison I can make is that this virtual monitoring, in detriment of the pandemic, right, which started because of the pandemic, facilitated our arrival to these women. So, we end up, -these women, these children and adolescents-, we end up having a greater ease of communication, a greater ease of dialogue, from the virtual environment.” - Brazil_ Boa Vista_#ID09_211019_111602-PRONTO |

Note that some codes were used for organizational purposes only and thus do not have specific quote experts, as they aggregated all child codes. For example, *Violence Type* is one such organizational code.
